# Epidemiologic study of odontogenic and non-odontogenic cysts in children and adolescents of a Brazilian population

**DOI:** 10.4317/medoral.22138

**Published:** 2017-12-24

**Authors:** Leorik-Pereira da Silva, Amanda-Katarinny-Goes Gonzaga, Mara-Luana-Batista Severo, Caio-César-da Silva Barros, Ana-Miryam-Costa de Medeiros, Lélia-Batista de Souza, Éricka-Janine-Dantas da Silveira

**Affiliations:** 1 Postgraduate Program in Oral Pathology, Dentistry Department, Federal University of Rio Grande do Norte, Natal, RN, Brazil

## Abstract

**Background:**

The objective of this study was to describe the frequency of cystic lesions in a Brazilian population of patients histopathologically diagnosed in the first and second decade of life.

**Material and Methods:**

Retrospective descriptive cross-sectional study was performed. Biopsy records were obtained from the archives of a Brazilian referral center between 1980 and 2016.

**Results:**

A total of 2.114 biopsy records of pediatric patients were analyzed with oral and maxillofacial lesions. Data such as gender, age, anatomical location, and histopathological diagnosis were collected and categorized. Among all oral and maxillofacial lesions (n=2.114), were diagnosed 294 cases of odontogenic cysts (13.9%) and 16 cases of non-odontogenic cysts (0.8%). The most frequent lesions in each group were, respectively: radicular cyst (n=145) and epidermoid cyst (n=4). These lesions were most common in female (n=158), with a mean age of 14 years. For intraosseous lesions, the mandible (n=148) was the most affected anatomic site; moreover, the floor of the mouth (n=6) was most affected by cysts in soft tissues.

**Conclusions:**

Odontogenic cysts were relatively common in population studied, but non-odontogenic cysts were rare in these patients.

** Key words:**Odontogenic cysts, non-odontogenic cysts, cysts in children, cysts in adolescents.

## Introduction

Different types of lesions can affect the maxillofacial complex of children and adolescents, which range from benign and indolent lesions to malignant tumors with aggressive behavior. In some situations, the symptoms of these lesions differ from those observed in adults (1,2).

The estimated incidence of maxillofacial lesions in the pediatric population ranges from 7-15% depending on the age range analyzed in each study and the region where the survey was conducted (1-4). Knowledge of epidemiological data of oral lesions in children and adolescents is important for a better understanding of lesions that can affect this population, contributing to a correct diagnosis, early treatment and favorable prognosis (1,2).

Odontogenic cysts arise from remnants of the odontogenic epithelium entrapped in bone or gingival tissue, while non-odontogenic cysts develop from epithelium of non-odontogenic origin. These lesions generally show slow and expansive growth and are associated in some cases with marked bone destruction and recurrence (3,5).

Despite the large number of studies on odontogenic and non-odontogenic cysts, data about the demographic profile of these lesions in children and adolescents are sparse. Therefore, the objective of this study was to describe the frequency of cystic lesions in a Brazilian population of patients histopathologically diagnosed in the first and second decade of life.

## Material and Methods

The study was approved by the local Ethics Committee (Approval No. 1.768.092). The biopsies and histopathological records obtained from the archives of a referral center for oral diagnostics in Brazil, were evaluated in Oral Pathology Service of the Federal University of Rio Grande do Norte (northeastern region).

In a retrospective study (1980-2016) a total of 2.114 cases of patients aged ≤ 19 years were analyzed. Data such as gender, race, age, anatomical location and histopathological diagnosis were collected and analyzed. The cysts were categorized into: Odontogenic and non-odontogenic cysts according to the current classification of the World Health Organization (WHO) (6). Some cysts not presented by this classification were categorized according to the previous literature (5,7).

The data were analyzed by descriptive statistics, using the SPSS 17.0 software (Statistical Package for the Social Sciences, Chicago, IL, USA).

## Results

A total of 14,565 oral and maxillofacial lesions were diagnosed during the study period, 2,114 of them in children and adolescents ranging in age from 0 to 19 years. There were 310 cases of cysts involving the maxillofacial complex, corresponding to 14.7% of all lesions diagnosed in this age group. Of these, 294 cases (94.8%) were odontogenic cysts and 16 (5.2%) were non-odontogenic cysts.

Most cases (n = 266, 85%) were diagnosed in the second decade of life ( Table 1). The gender distribution was homogenous, with a slight predominance of both groups of cysts in females (n = 158, 51%) ( Table 2, Table 3).

**Table 1:**
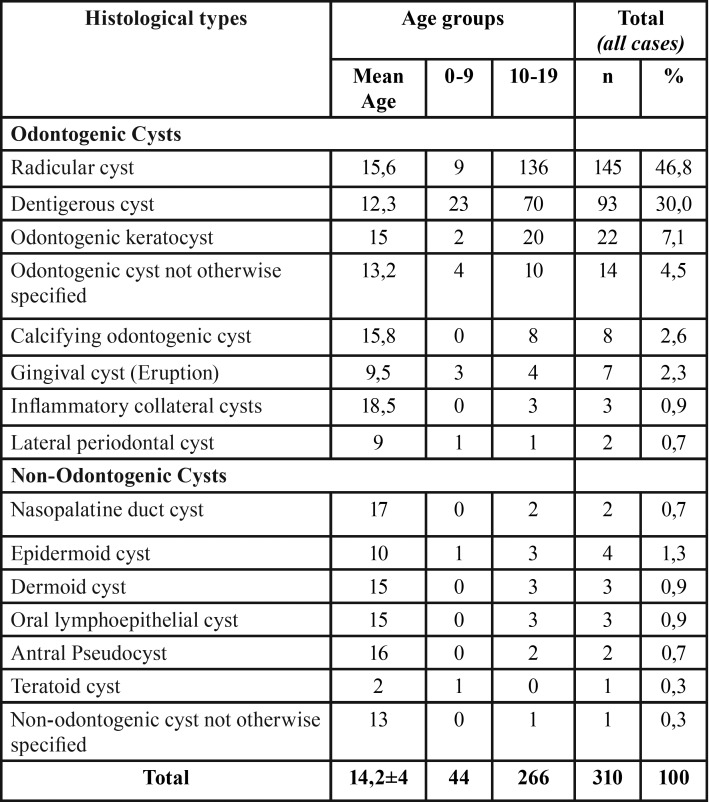
Age distribution of odontogenic and non-odontogenic cysts in children and adolescent population.

**Table 2:**
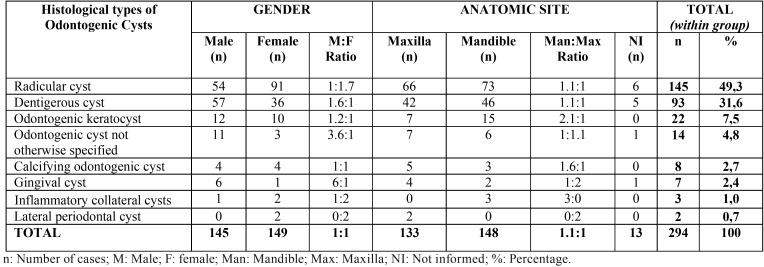
Gender and anatomic site of odontogenic cysts in children and adolescents.

**Table 3:**
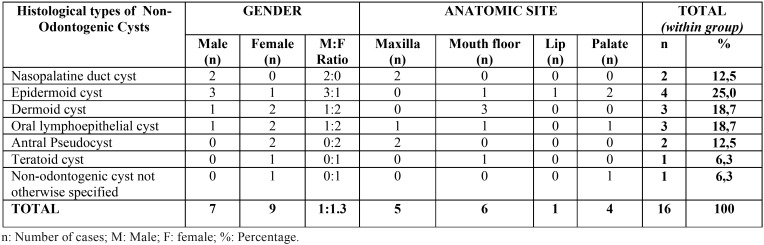
Gender and anatomic site of non-odontogenic cysts in children and adolescents.

The mandible was the site most commonly affected by odontogenic cysts (n = 148, 56%). Overall, radicular cysts were the most common lesion (n = 145, 49.3%). However, dentigerous cysts were the most frequent lesion in patients diagnosed between 0-9 years (n = 23, 52%) ( Table 2).

Non-odontogenic cysts were extremely rare in pediatric patients, especially in the first decade of life (n = 2), (8,9) The anatomical site most affected by these cysts was the floor of the mouth (n = 6 37%) and epidermoid cysts were the most prevalent (n = 4, 25%) ( Table 3).

## Discussion

Studies using histopathological data are fundamental to characterize and establish the true incidence of oral and maxillofacial diseases in different age groups (2,3). In this respect, research evaluating biopsy records is necessary to analyze the main types of lesions that occur in a given population and to provide data that can guide dentists and pediatricians particularly in the diagnosis and management of children and adolescents, since little is known about the prevalence of cystic lesions in this population group.

Patients aged 0 to 19 years were included in the present study. Similar studies in the literature involve a variety of age groups: 0 to 14 years (6), 0 to 16 years (2-4,10), and 0 to 19 years (1). Despite the heterogeneity in age classification, all of these groups are considered pediatric. In the present study, the range of 0 to 19 years was adopted since the objective was to emphasize the incidence of odontogenic and non-odontogenic cysts in early childhood (0 to 10 years) and adolescence (10-19 years) and thus to observe the distribution of these lesions at our diagnostic service in the pediatric age group.

The present study found 14.7% of children and adolescents diagnosed with oral lesions over a period of 36 years. Similar rates have been reported by Souza *et al.* (3) in Brazil (11%), Zuniga *et al.* (9) in Chile (20.6%), Lei *et al.* (10) in Taiwan (19.16%), Ha *et al.* (4) in Australia (18,5%), and Cavalcante *et al.* (2) also in Brazil (13.6%). On the other hand, lower rates of biopsy records in patients of this age range have been reported by Skiavounou *et al.* (11) in Greece (2.38%) and by Gultelkin *et al.* (12) in Turkey (5.5%). It is suggested that the higher percentages of oral diseases diagnosed in this study conducted in northeastern Brazil (14.7%), as well as in other countries, reflect the larger pediatric populations of these countries compared to Greece and Turkey (11,12).

We found a slight predominance of odontogenic and non-odontogenic cysts among female patients (51%), in agreement with other studies conducted in Brazil (1,2,3,7,8,13,14). A higher incidence among males has been reported in other regions of the world such as Italy, Taiwan, Australia, and Turkey (4,10,15,16). The slight female predilection of these lesions observed in the present study may be a particularity of the population of northeastern Brazil.

The mandible was the most commonly affected site in the population studied (47.74%), in agreement with the findings of Skiavounou et al. (11) and Johnson et al. (17), but differing from the results reported by Grossmann et al. (14), Souza *et al.* (3) and Demirkol *et al.* (16) who found a higher incidence in the maxilla.

Odontogenic cysts accounted for more than 90% of the cases analyzed here, in agreement with previous studies (1,2,3,8,12). Radicular cyst (46.8%), dentigerous cyst (30%) and odontogenic keratocyst (7.1%) were the most prevalent odontogenic cysts. Similar results have been reported by Gultelkin *et al.* (12), Grossmann *et al.* (14), Pessoa *et al.* (1) and Cavalcante *et al.* (2). However, dentigerous cysts were the most prevalent in the first decade of life (0 to 9 years).

Some studies have shown radicular cysts to be the most frequent in the age range of 0 to 19 years (2,12). These data may in part reflect the poor oral health conditions of the pediatric populations analyzed since radicular cyst is a lesion associated with odontogenic infections due to caries progression. In this respect, actions promoting oral health in children and adolescents are important to reduce the prevalence of these inflammatory lesions.

Dentigerous cysts were also commonly diagnosed in this age group considering their association with impacted third molars and upper canines that erupt around the second decade of life (1,2,12). Some authors suggested that the spread of inflammatory exudate from an infected primary tooth within the dental follicle of an impacted permanent tooth could give rise to an inflammatory dentigerous cyst; however, in this case, the term “inflammatory follicular cyst” seems more appropriate (13). On the other hand, in the present study all dentigerous cysts with or without inflammation were classified in the same group due to the histopathological similarities of the cystic lining.

A variable frequency of cases of odontogenic keratocyst in the pediatric population has been reported in the literature (1.2% to 6%) (8,12). Odontogenic keratocyst represent a clinical challenge since they require an early diagnosis and individualized treatment because of their higher aggressiveness and potential of recurrence, facts that may lead to radical and often mutilating surgical removal (1,3,8,12).

Other odontogenic cysts such as calcifying odontogenic cyst, gingival cyst, inflammatory collateral cysts and lateral periodontal cyst were less frequent, corroborating other epidemiological surveys (1,3,5,8,9).

According to the WHO’s recent classification (6), vestibular bifurcation cysts and paradental cyst are inflammatory odontogenic cysts that have been included in a single category called inflammatory collateral cysts, to simplify the nomenclature, since the treatment of these lesions remains the same. In the present study, no case of buccal bifurcation cyst was identified. We believe that this clinical diagnosis really is common in this age group. However, because of lack of precise clinical data these cysts must have been probably histopathologically diagnosed as odontogenic cysts without other specification, this fact does not change the therapeutics of these cases.

Non-odontogenic cysts were rare in the population studied and epidermoid cysts were the most prevalent (1.3%), in agreement with the studies of Zuniga *et al.*, (9), Lei *et al.* (10), and Pêssoa *et al.* (1). On the other hand, Nonaka *et al.* (5), Johnson *et al.* (17) and Vasconcelos *et al.* (7) identified a higher frequency of nasopalatine duct cysts. Some studies reported a higher frequency of non-odontogenic cysts in the cheek mucosa, followed by the maxilla (5,17). In contrast, in the present study the floor of the mouth and maxilla were the most commonly affected sites.

Like in the present series, other studies report the histopathological diagnosis of cystic lesions without other specifications (1-3). This diagnostic difficulty is believed to be due to the lack of clinical and radiographic data, which impairs a conclusive diagnosis of cystic lesions of the maxillofacial complex.

## Conclusions

In conclusion, among all cystic lesions evaluated, dentigerous cysts were the most common in children (0 to 9 years) and radicular cysts in adolescents (9 to 19 years). This high incidence of radicular cysts may be attributed to the precarious oral conditions of the population studied and the lack of public incentives to prevent oral infectious diseases. We emphasize that studies using histopathological data can provide important information about the incidence of oral and maxillofacial lesions.
